# Medical Society of the County of Erie

**Published:** 1903-12

**Authors:** Franklin C. Gram


### Medical Society of the County of Erie.

*Special Meeting, November 9, 1903.*

Reported by FRANKLIN C. GRAM, M. D., Secretary.

AT 4.30 p. m. on Monday, November 9, 1903, the Medical Society of the County of Erie assembled in special session in the rooms of the Buffalo Society of Natural Sciences. The president, Dr. Ernest Wende, announced that the object of the call was to take appropriate action on the death of Dr. C. C. Wyckoff, which occurred November 7, 1903.

On motion of Dr. J. B. Coakley, the president appointed a committee, consisting of Drs. J. B. Coakley, Lucien Howe, Henry R. Hopkins, D. W. Harrington and Frederick F. Hoyer, to prepare a suitable expression of regard for the deceased.

The committee presented the following memorial:

IN MEMORIAM.

Cornelius C. Wyckoff.

As we meet to mourn the loss of one of our oldest and most respected associates we wish to express our affectionate regard in some tribute to his memory. As a man he always has been identified with what was elevating and of good repute, active in the interests of the community, and always foremost in his efforts to make the city better and purer and more beautiful.

Dr. Wyckoff was one of the builders of the city, in all that pertains to the medical profession. We honor the work which he has done in assisting in the foundation of its largest hospital, in giving his services gratuitously to it for a large part of his long life, and in identifying himself also, with the interests of other institutions of a similar kind.. We admire, too, his bravery in the days of cholera when he faced death in all the forms which that dread pestilence presented.

Most of all, we cherish the beautiful example he has left as an earnest, quiet and conscientious physician. His life is a record of nameless unremembered acts of kindness and of love. These are graven deep in the hearts of a large circle of patients and friends among whom he has lived for more than half a century. His example serves as a model of the good and true physician, and it is with affectionate remembrances that we spread upon the records of this society this imperfect tribute to the memory of our beloved associate, Cornelius C. Wyckoff.

J. B. Coakley,
F. F. Hoyer,
H. R. Hopkins,
D. W. Harrington,
Lucien Howe.

Brief and feeling tributes were paid by many of the physicians present.

Dr. Thomas M. Johnson said that when he began the practice of medicine in 1861, there were about fifty-two regular practitioners in Buffalo, and all were members of this society. Of this number only Dr. John Hauenstein and the speaker remain. Dr. Wyckoff was a member for about forty-six years. He had a large practice, was always active, careful and a typical general practitioner.

Dr. William C. Phelps had known the deceased since 1862 and was much associated with him in practice. He kept clear of most medical dissensions, which were bitter in the earlier periods. He held the confidence of his patients and enjoyed remarkable health and activity throughout his long career.

Dr. William Warren Potter said it is vouchsafed to but few men to live the life or die the death of this physician, whose taking off we now lament. Dr. Wyckoff as a physician had the respect and love of his patients, as a citizen he performed his duty in the community in which he lived, and as a man he represented all that could be desired by the idealist. He regularly attended the meetings of this society, and his familiar figure will be missed by every member.

Dr. Conrad Diehl knew him since 1862, and as a colleague since 1866. He spoke of his professional courtesy towards his fellow practitioners.

Dr. J. B. Coakley described the beautiful tributes paid Dr. Wyckoff by his patients in their evidences of love and affection, and told of his personal loss as a friend.

Dr. Lucien Howe called attention to the large gathering of physicians who had come to this meeting by a common impulse to pay their tribute and show their respect.

Dr. Charles S. Jewett spoke for the younger members of the profession, and alluded to his recent intimate association with Dr. Wyckoff at the Home for Old Ladies.

Dr. Frederick F. Hoyer said Dr. Wyckoff was his oldest friend in the medical profession, having known him for over fifty years. He emphasised his personal loss.

Dr. Wm. C. Krauss said that the one thing that impressed him most was the fact that Dr. Wyckoff grew older gracefully. Even in old age he seemed the embodiment of youth.

Dr. D. W. Harrington stated that Dr. Wyckoff was the first physician appointed on the staff of the Buffalo General Hospital in 1858, remaining on the regular staff until a recent date when he was transferred to the consulting staff.

Dr. Henry R. Hopkins said it was easy to be calm in prosperity but difficult in adversity. The deceased met the most serious test with the highest possible dignity and manhood.

Dr. A. H. Briggs related incidents of the last meeting with his departed friend.

Dr. Irving M. Snow remembered Dr. Wyckoff from early youth as having made his calls on horseback, a custom which he continued to the end of life. His age seemed to come gradually.

Dr. Wetmore added a brief tribute after which Dr. Krauss suggested that members meet at his office and attend the funeral of Dr. Wyckoff in a body.

